# Emerging research themes in maternal hypothyroidism: a bibliometric exploration

**DOI:** 10.3389/fimmu.2024.1370707

**Published:** 2024-03-26

**Authors:** Ailing Chen, Zouqing Luo, Jinqiu Zhang, Xiaohui Cao

**Affiliations:** ^1^Research Institute for Reproductive Health and Genetic Diseases, Women’s Hospital of Jiangnan University, Wuxi Maternity and Child Health Care Hospital, Wuxi, China; ^2^Department of Obstetrics, Women’s Hospital of Jiangnan University, Wuxi Maternity and Child Health Care Hospital, Wuxi, China; ^3^Department of Pathology, Women’s Hospital of Jiangnan University, Wuxi Maternity and Child Health Care Hospital, Wuxi, China

**Keywords:** maternal hypothyroidism, bibliometrics, CiteSpace, VOSviewer, visualization, emerging topics, research focus

## Abstract

**Background:**

Hypothyroidism, a prevalent endocrine disorder, carries significant implications for maternal and infant health, especially in the context of maternal hypothyroidism. Despite a gradual surge in recent research, achieving a comprehensive understanding of the current state, focal points, and developmental trends in this field remains challenging. Clarifying these aspects and advancing research could notably enhance maternal-infant health outcomes. Therefore, this study employs bibliometric methods to systematically scrutinize maternal hypothyroidism research, serving as a reference for further investigations.

**Objective:**

Through bibliometric analysis, this study seeks to unveil key research focus areas, developmental trends, and primary contributors in Maternal Hypothyroidism. The findings offer insights and recommendations to inform future research endeavors in this domain.

**Methods:**

Literature metrics analysis was performed on data retrieved and extracted from the Web of Science Core Collection database. The analysis examined the evolution and thematic trends of literature related to Maternal Hypothyroidism. Data were collected on October 28, 2023, and bibliometric analysis was performed using VOSviewer, CiteSpace, and the Bibliometrix software package, considering specific characteristics such as publication year, country/region, institution, authorship, journals, references, and keywords.

**Results:**

Retrieved from 1,078 journals, 4,184 articles were authored by 18,037 contributors in 4,580 institutions across 113 countries/regions on six continents. Maternal Hypothyroidism research publications surged from 44 to 310 annually, a 604.54% growth from 1991 to 2022. The USA (940 articles, 45,233 citations), China Medical University (82 articles, 2,176 citations), and Teng, Weiping (52 articles, 1,347 citations) emerged as the most productive country, institution, and author, respectively. “Thyroid” topped with 233 publications, followed by “Journal of Clinical Endocrinology & Metabolism” (202) with the most citations (18,513). “Pregnancy” was the most cited keyword, with recent high-frequency keywords such as “outcome,” “gestational diabetes,” “iodine intake,” “preterm birth,” “guideline,” and “diagnosis” signaling emerging themes in Maternal Hypothyroidism.

**Conclusions:**

This study unveils developmental trends, global collaboration patterns, foundational knowledge, and emerging frontiers in Maternal Hypothyroidism. Over 30 years, research has predominantly focused on aspects like diagnosis, treatment guidelines, thyroid function during pregnancy, and postpartum outcomes, with a central emphasis on the correlation between maternal and fetal health.

## 1 Introduction

Hypothyroidism is an endocrine system disorder primarily caused by insufficient secretion of thyroid hormones. Dominantly, primary hypothyroidism is characterized by elevated thyroid-stimulating hormone (TSH) levels, accompanied by Free Thyroxine 4 (FT4) concentrations below the reference range. Subclinical hypothyroidism, considered an early sign of thyroid dysfunction, is characterized by elevated TSH levels while FT4 concentrations remain within the reference range (1). Multiple factors, including iodine deficiency (2), Hashimoto’s thyroiditis (3), and rare causes such as congenital, drug-related, iatrogenic, and infiltrative diseases, may contribute to the development of hypothyroidism (1).

During pregnancy, hormonal fluctuations may impact thyroid function in pregnant women. Factors such as the rise in human chorionic gonadotropin, increased synthesis and secretion of thyroxine-binding globulin by the liver (4), and enhanced renal clearance of iodine (5) can influence maternal thyroid function. Maternal hypothyroidism often presents as subclinical hypothyroidism, with most patients lacking significant clinical symptoms. However, a minority may exhibit clinical hypothyroidism or isolated low thyroxine levels (6). The prevalence of hypothyroidism during pregnancy varies from 0.5% to 3.47% (7, 8), highlighting its relatively high incidence among pregnant women and establishing it as one of the common chronic conditions during pregnancy (9).

Maternal hypothyroidism increases the risk of complications during pregnancy, including gestational hypertension (10), preeclampsia (11), gestational diabetes (12), and preterm birth (13). Thyroid hormones play a crucial role in fetal neurological and intellectual development. Maternal hypothyroidism during pregnancy may elevate the risk of intellectual developmental defects (14), preterm birth, low birth weight (15), and fetal hypothyroidism (16). Therefore, maintaining optimal thyroid function is crucial for pregnant women.

Bibliometric analysis, a systematic method aimed at evaluating, summarizing, and interpreting a large body of literature, provides insights into research trends, hotspots, and developmental directions in a specific field or topic (17). The analysis combines principles of statistics and informatics to quantify and assess literature resources, revealing characteristics of a particular research area. Maternal Hypothyroidism has been a subject of clinical and scientific interest. Despite existing reviews focusing on different aspects of Maternal Hypothyroidism (18, 19), bibliometric analysis offers a more comprehensive and intuitive understanding of its development and trends.

This paper aims to conduct a bibliometric analysis using tools such as CiteSpace and VOSviewer on publications related to Maternal Hypothyroidism in the Web of Science Core Collection (WoSCC). The analysis encompasses aspects such as the annual distribution of publications, countries, institutions, authors, source journals, keyword co-occurrence, and reference co-occurrence. Through this analysis, the goal is to gain in-depth insights into the current state, focal areas, and future trends of research on Maternal Hypothyroidism. This study will assist new researchers and experts in delineating the research scope, identifying novel topics, or planning future research directions, thereby providing guidance for both clinical practice and scientific inquiry.

## 2 Methods

### 2.1 Data collection and retrieval strategy

**Figure 1:**
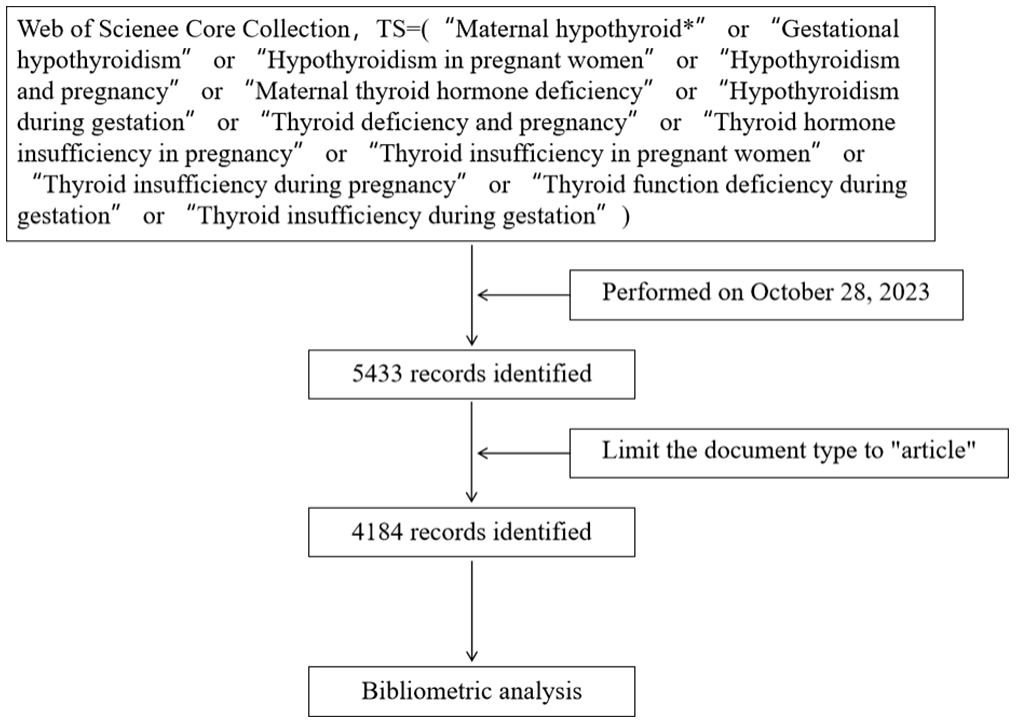
Flowchart - Maternal Hypothyroidism search process.

To enhance the representativeness and accessibility of the data, a literature search was conducted within the WoSCC on October 28, 2023, and the data collection and retrieval strategy is illustrated in **Figure 1**. The search terms, identified through TS (“topic”, covering title, abstract, author keywords, and keywords plus), were set as TS=(“Maternal hypothyroid*” or “Gestational hypothyroidism” or “Hypothyroidism in pregnant women” or “Hypothyroidism and pregnancy” or “Maternal thyroid hormone deficiency” or “Hypothyroidism during gestation” or “Thyroid deficiency and pregnancy” or “Thyroid hormone insufficiency in pregnancy” or “Thyroid insufficiency in pregnant women” or “Thyroid insufficiency during pregnancy” or “Thyroid function deficiency during gestation” or “Thyroid insufficiency during gestation”). The publication type was limited to articles, with no restrictions imposed on time and language. A total of 4184 records were obtained, collecting information on publications, authors, countries, institutions, journals, keywords, and citations, and were exported in the form of complete citation records.

### 2.2 Data analysis

Literature metrics data analysis was performed using VOSviewer (v1.6.19), CiteSpace (v.6.1.R6 Advanced), and the bibliometrics software package (version 4.1.3) based on the R language (4.3.1). Preliminary descriptive statistics of the number of publications and citations by year, country and author were performed using the “Analyze” function in WoSCC. The bibliometrics package was used to visualize publication distribution and collaboration between countries/regions. VOSviewer was used for data extraction and visualization of countries, institutions, authors and keywords. CiteSpace was used to construct dual map overlays of journals related to Maternal Hypothyroidism and visualizations of keyword timelines and cluster maps, detecting the top 25 bursty keywords.

## 3 Results

### 3.1 Annual global publication outputs on Maternal Hypothyroidism

**Figure 2:**
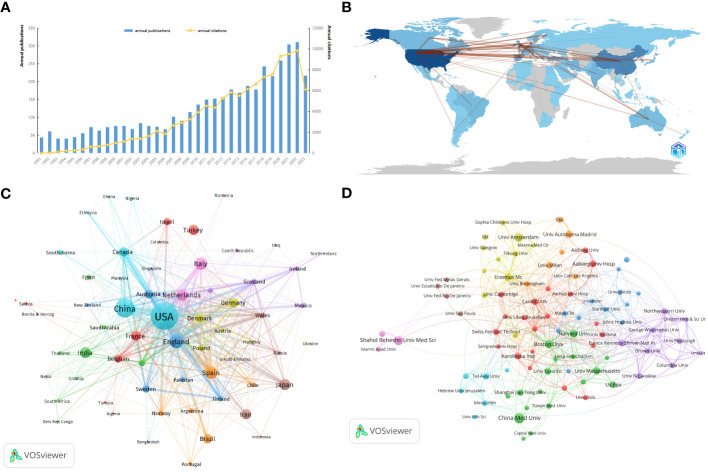
The distribution of Maternal Hypothyroidism publications over time and collaboration networks among countries/regions/institutions related to the subject. **(A)** Distribution of Maternal Hypothyroidism publications over time. **(B)** Distribution and collaboration of publications among countries/regions. **(C)** Collaboration clusters among countries/regions. **(D)** Visualization map of institutional collaboration.

The number of publications over time is indicative of research trends and progress in a given field. According to records from the WoSCC database, literature on Maternal Hypothyroidism stretches back to 1991, with 4184 articles identified in total. **Figure 2A** charts the yearly publication output concerning “Maternal Hypothyroidism,” with an inaugural count of 44 in 1991. This suggests the early initiation and development of research in this domain. The literature in this field has undergone consistent growth, with an annual growth rate of 5.11%. The overall global annual publication output increased steeply from a meager 44 articles in 1991 to an astonishing 310 articles in 2022, a remarkable 604.54% growth. From 1991 to 2006, the annual publication count remained below 100 articles. However, between 2009 and 2017, the number of publications progressively increased from 115 to 178. In 2018, the publication count surmounted the 200 mark, ultimately exceeding 300 by 2022. The total number of citations for publications collected from 1991 to 2023 was 114,378, with an average of 27.34 citations per article. The consistent increase in citations every year signifies continual research attention towards “Maternal Hypothyroidism” during the past 30 years.

### 3.2 Distribution and co-authorship of countries/regions

Maternal Hypothyroidism is a globally recognized research focus. Between 1991 and 2023, 113 countries/regions across six continents contributed to the study of Maternal Hypothyroidism. The Countries’ Collaboration World Map in **Figure 2B** illustrates close collaboration between North America, East Asia, and Western Europe. **Table 1** highlights the top 10 most productive countries, showcasing the USA (940 publications, 45233 citations) as the most prolific nation, constituting 22.47% of the total publications. China (471 publications, 6614 citations) follows at 11.26%, and the England (263 publications, 12571 citations) closely trails, representing 6.29% of the total publications. These three countries collectively contribute over 40% of the research output, underscoring a high level of interest in Maternal Hypothyroidism research.

**Table 1:** Top 10 most productive countries in Maternal Hypothyroidism research.

Rank	Country	Publications n(%)	Total citations	Average citations	H-Index	Total link strength
1	USA	940(22.47)	45233	48.12	94	471
2	China	471(11.26)	6614	14.04	41	157
3	England	263(6.29)	12571	47.8	54	302
4	Spain	222(5.31)	8227	37.06	46	160
5	Italy	214(5.12)	7824	36.56	46	104
6	Netherlands	190(4.54)	10751	56.58	53	216
7	Japan	185(4.42)	4888	26.42	32	71
8	India	166(3.97)	1739	10.48	20	32
9	Iran	151(3.61)	2028	13.43	25	31
10	Germany	148(3.54)	3412	23.05	28	97

VOSviewer analyzed collaborative authors among countries/regions, utilizing the parameters: Method (Association Strength) and a minimum number of country documents: 5. From the results involving 113 countries, 67 met the threshold. Netherlands, the USA, and England exhibited the highest Total link strength, Average citations, and H-Index (**Table 1**), indicating their relatively mature research outcomes in Maternal Hypothyroidism. These nations showcase more significant connections, collaborations, and influence compared to others.

Based on co-authorship analysis, VOSviewer categorized countries into different clusters. The visual representation in **Figure 2C** depicts the collaborative author network for Maternal Hypothyroidism, divided into nine clusters. The light blue cluster comprises 7 countries, centered around the USA, China, and Canada. The blue cluster includes countries such as England, Australia, Sweden, and Finland. The orange cluster consists of Spain, Brazil, Norway, and Argentina among others. The pink cluster includes Italy, the Netherlands, and Romania. Japan frequently collaborates with Iran, Wales, and Russia (brownish). India closely collaborates with Switzerland, Saudi Arabia, Thailand, and other countries (green). The red cluster comprises Turkey, France, Belgium, Israel, Serbia, and other nations. Germany closely collaborates with Denmark, Poland, Austria, and other countries (yellowish-green).

### 3.3 Distribution of research institutions and authors

#### 3.3.1 Distribution of research institutions

Utilizing VOSviewer, cooperative author analysis was conducted on these institutions, with parameters set as Method (Association Strength) and a minimum number of documents: 15. The results, drawn from 4580 institutions, identified 90 institutions meeting the threshold. **Table 2** showcases the top 10 most productive institutions, where China Medical University (n=82, 1.96%) emerges as the institution with the highest publication output, followed by Shahid Beheshti University of Medical Sciences (n=68, 1.63%), Universiteit van Amsterdam (n=59, 1.41%), Boston University (n=49, 1.17%), and Harvard University (n=46, 1.10%). Among the top 10 institutions, three are located in the USA. Universidad Autonoma de Madrid (95.32) boasts the highest Average citations, followed by Harvard University (87.83) and University of Massachusetts (74.08). Erasmus Medical Center leads in Total link strength (79) among the top 10 institutions, followed by Boston University (53) and Aalborg Universitets Hospital (47), indicating these institutions maintain closer connections with others in Maternal Hypothyroidism research.

**Table 2:** Top 10 most productive research institutions in Maternal Hypothyroidism research.

Rank	Institution	Country	Publications n (%)	Total citations	Average citations	Total link strength
1	China Medical University	China	82(1.96)	2176	26.54	10
2	Shahid Beheshti University of Medical Sciences	Iran	68(1.63)	1045	15.37	34
3	Universiteit van Amsterdam	Netherlands	59(1.41)	3710	62.88	27
4	Boston University	USA	49(1.17)	2918	59.55	53
5	Harvard University	USA	46(1.1)	4040	87.83	39
6	Erasmus Medical Center	Netherlands	45(1.08)	3215	71.44	79
7	Universidad Autonoma de Madrid	Spain	41(0.98)	3908	95.32	24
8	University of Massachusetts	USA	40(0.96)	2963	74.08	22
9	Karolinska Institutet	Sweden	36(0.86)	587	16.31	29
10	Aalborg Universitets Hospital	Denmark	35(0.84)	1873	53.51	47

**Figure 2D** categorizes leading publishing institutions into nine clusters. The red cluster comprises primarily European institutions, including Karolinska Inst, Aalborg Univ Hosp, and Cardiff Univ. The green cluster is dominated by institutions from China, the USA, and Canada, such as China Med Univ, Boston Univ, Harvard Univ, and Univ Massachusetts. The blue cluster consists mainly of US institutions, including Mayo Clin, Univ Illinois, and Stanford Univ. Yellow cluster is primarily composed of Netherlands institutions, such as Univ Amsterdam, Erasmus Mc, and Tilburg Univ. The purple cluster is made up of US institutions, including Eunice Kennedy Shriver Natl Inst Child Hlth & Hum, Univ N Carolina, and Northwestern Univ. The light blue cluster is predominantly composed of Israeli institutions, including Tel Aviv Univ, Ben Gurion Univ Negev, and Hebrew Univ Jerusalem. The orange cluster is mainly European institutions, including Univ Autonoma Madrid, Univ Milan, Csic, and Univ Birmingham. The brown cluster is dominated by Brazilian institutions, such as Univ Sao Paulo, Univ Fed Rio De Janeiro, and Univ Estado Rio De Janeiro. The relatively distant pink cluster is comprised of Iranian institutions, such as Shahid Beheshti Univ Med Sci, Univ Tehran Med Sci, and Islamic Azad Univ.

#### 3.3.2 Author distribution

The most prolific author is Teng, Weiping (China Medical University) with 52 publications (1.24%), followed by Shan, Zhongyan (China Medical University) with 51 publications (1.22%) and Azizi, Fereidoun (Shahid Beheshti University Medical Sciences) with 50 publications (1.2%) (**Table 3**). Among the top 10 most productive authors, the highest Average citations are attributed to Lazarus, John H., Laurberg, Peter, and Peeters, Robin P. The authors with the highest H-Index are Peeters, Robin P., and Visser, Theo J. Teng, Weiping, Shan, Zhongyan, and Peeters, Robin P. exhibit the highest Total link strength, indicating close collaboration with other authors in Maternal Hypothyroidism research.

**Table 3:** Top 10 most productive authors in Maternal Hypothyroidism research.

Rank	Author	Institution	Publications n(%)	Total citations	Average citations	H-index	Total link strength
1	Teng, Weiping	China Medical University	52(1.24)	1347	25.9	17	213
2	Shan, Zhongyan	China Medical University	51(1.22)	1328	26.04	17	190
3	Azizi, Fereidoun	Shahid Beheshti University Medical Sciences	50(1.2)	1022	20.44	17	56
4	Pearce, Elizabeth N.	Boston Univ Chobanian	46(1.1)	3089	67.15	22	40
5	Korevaar, Tim I. M.	Erasmus University Medical Center	38(0.91)	1731	45.55	22	158
6	Peeters, Robin P.	Erasmus University Medical Center	38(0.91)	3289	86.55	24	187
7	Lazarus, John H.	Cardiff University	35(0.84)	4060	116	21	14
8	Visser, Theo J.	Erasmus University Medical Center	31(0.74)	2233	72.03	24	140
9	Andersen, Stine Linding	Aalborg University Hospital	29(0.69)	561	19.34	13	27
10	Laurberg, Peter	Aalborg University Hospital	29(0.69)	3242	111.79	20	23

**Figure 3:**
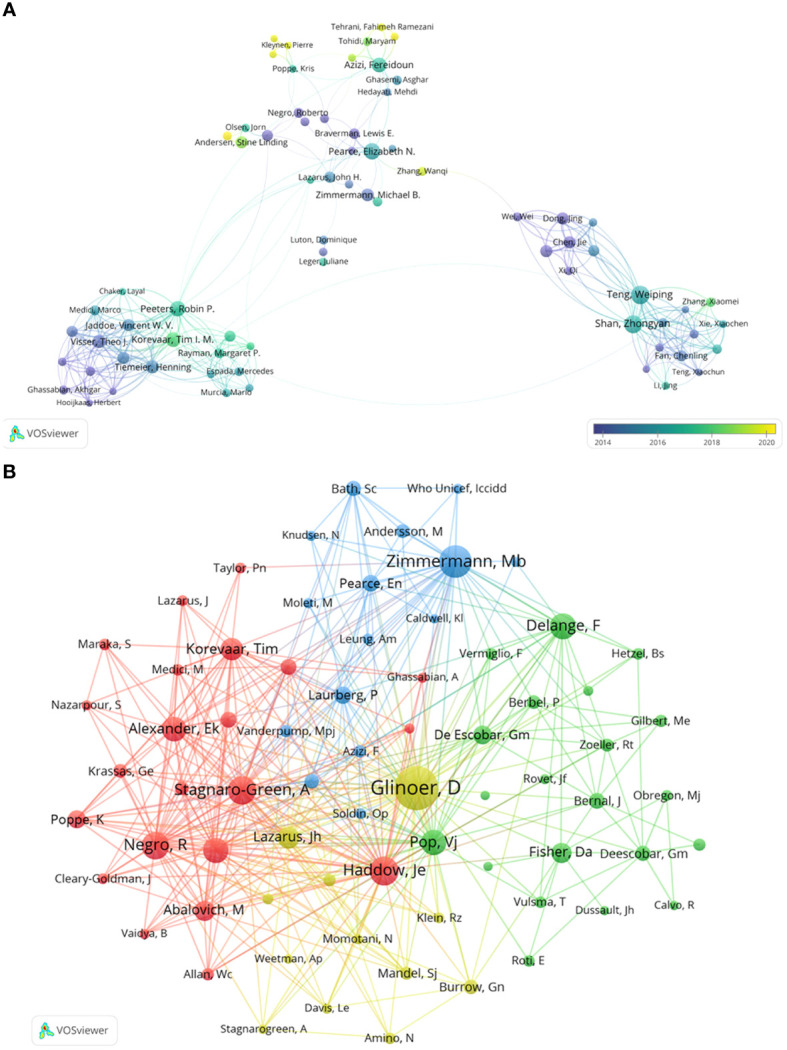
Collaboration networks among authors in Maternal Hypothyroidism. **(A)** Visualization map of author collaboration. **(B)** Co-citation analysis of cited authors.

Further collaborative author analysis using VOSviewer, with parameters set as Method (Association Strength) and the minimum number of documents: 9, identified 113 authors from 18037 results who met the threshold. Based on co-authorship frequency and density, authors were categorized into clusters, with overlay visualization using publication timelines for color annotation. **Figure 3A** depicts each node as an author, with circle size reflecting the number of articles published, and connecting lines representing co-occurrence relationships. Different clusters represent collaborations between authors. Notably, Teng, Weiping collaborates closely with Shan, Zhongyan; Fan, Chenling; Li, Chenyan; Zhang, Xiaomei. Peeters, Robin P. collaborates closely with Tiemeier, Henning; De Rijke, Yolanda B.; Korevaar, Tim I.M.; Jaddoe, Vincent W.V. Pearce, ElizabethN. collaborates closely with Braverman, Lewis E.; Laurberg, Peter; Lazarus, John H. The yellow nodes in **Figure 3A** represent authors who have remained consistently active and continually contributed to papers in recent times, such as Andersen, Stig; Nazarpour, Sima; Kleynen, Pierre; Rozenberg, Serge; Veltri, Flora; Tehrani, Fahimeh, Ramezani; Zhang, Wanqi.

We conducted a co-citation analysis of cited authors to gain deeper insights into the intrinsic connections and knowledge structure among the literature. **Figure 3B** depicts the citation patterns of prominent researchers on the topic of Maternal Hypothyroidism. The top 5 most cited authors are Glinoer, Daniel; Zimmermann, Mb; Haddow, Je; Stagnaro-Green, A; and Negro, R. These authors hold significant positions within the co-citation network and collaborate closely. Their citation counts far exceed those of other authors, demonstrating their widespread influence and core status within the research field.

### 3.4 Discipline and journal distribution

#### 3.4.1 Disciplines

In terms of the quantity of published papers, Endocrinology Metabolism (36.59%), Obstetrics Gynecology (14.91%), and Medicine General Internal (10.37%) are the top three disciplines (**Table 4**). Notably, over one-third of papers are published in Endocrinology Metabolism (36.59%). Other disciplines include Pediatrics (9.80%), Nutrition Dietetics (6.02%), Neurosciences (4.42%), among others.

**Table 4:** Top 10 disciplinary categories in Maternal Hypothyroidism research.

Rank	Web of Science Categories	Publications n(%)
1	Endocrinology Metabolism	1531(36.59)
2	Obstetrics Gynecology	624(14.91)
3	Medicine General Internal	434(10.37)
4	Pediatrics	410(9.80)
5	Nutrition Dietetics	252(6.02)
6	Neurosciences	185(4.42)
7	Public Environmental Occupational Health	185(4.42)
8	Toxicology	162(3.87)
9	Reproductive Biology	146(3.49)
10	Biochemistry Molecular Biology	137(3.27)

#### 3.4.2 Journal distribution

**Figure 4:**
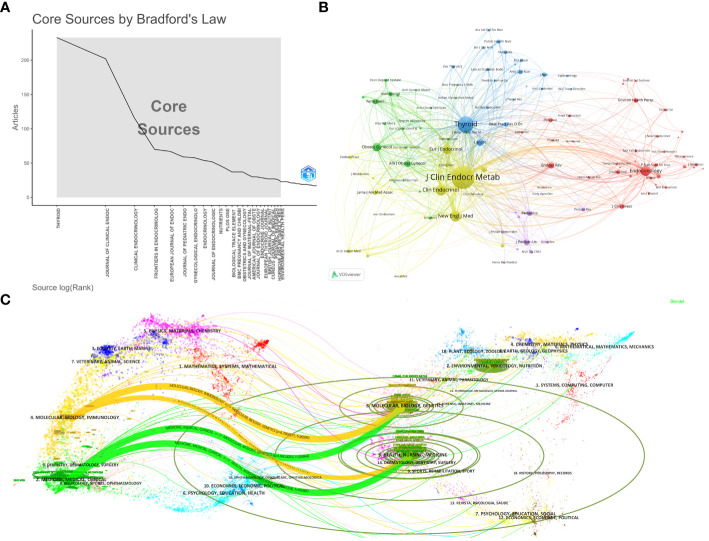
Analysis of journal sources. **(A)** The distribution of journal sources according to Bradford’s Law. **(B)** Co-citation analysis of cited sources regarding Maternal Hypothyroidism. **(C)** Dual-Map overlay of journals publishing research on Maternal Hypothyroidism.

The identified literature on Maternal Hypothyroidism was published in 1078 journals. Using Bradford’s Law, 24 journals were identified as core journals (**Figure 4A**). **Table 5** lists the top 10 journals publishing the most papers on Maternal Hypothyroidism, accounting for 22.90% (958/4184) of the total published papers. “Thyroid” tops the list with 233 papers, followed by “Journal of Clinical Endocrinology & Metabolism” with 202 papers and “Clinical Endocrinology” with 116. “Journal of Clinical Endocrinology & Metabolism” leads in citation count (n=18513), average citation count (91.65), and H-Index, making it the most influential journal. This can also be understood through the co-citation bibliometric map generated by VOSviewer, as shown in **Figure 4B**. The strength of connections between journals is typically represented by the thickness or darkness of the links. The thicker or darker the link, the closer the connection between two journals, indicating a higher number of co-citations between them. This suggests that these journals may exhibit higher similarity or complementarity in terms of research topics, methodologies, and knowledge dissemination. The co-citation analysis of cited sources indicates that the top three journals with the highest citations are the Journal of Clinical Endocrinology & Metabolism, Thyroid, and Clinical Endocrinology.

**Table 5:** Top 10 Journals in Maternal Hypothyroidism Research.

Rank	Journal	Publications n(%)	Total citations	Average citations	H-index	2022 JCR category quartile	2022 IF
1	Thyroid	233(5.57)	10907	46.81	52	Q1	6.6
2	Journal of Clinical Endocrinology & Metabolism	202(4.83)	18513	91.65	73	Q1	5.8
3	Clinical Endocrinology	116(2.77)	4467	38.51	30	Q3	3.2
4	Frontiers in Endocrinology	70(1.67)	348	4.97	10	Q1	5.2
5	European Journal of Endocrinology	67(1.6)	3552	53.01	32	Q1	5.8
6	Journal of Pediatric Endocrinology & Metabolism	59(1.41)	656	11.12	15	Q4	1.4
7	Gynecological Endocrinology	58(1.39)	555	9.57	12	Q4	2
8	Endocrinology	54(1.29)	3201	59.28	30	Q2	4.9
9	Journal of Endocrinological Investigation	52(1.24)	933	17.94	19	Q1	5.4
10	Nutrients	47(1.12)	455	9.68	13	Q1	5.9

**Figure 4C**, a dual-map overlay of journals, reveals the distribution of topics. Citing journals are on the left, cited journals on the right. Labels indicate the disciplines covered by the journals, and colored paths represent citation relationships. Four major paths stand out. The orange and green citation paths suggest that research from Molecular, Biology, Genetics journals and Health, Nursing, Medicine journals is often cited by Molecular/Biology/Immunology journals or Medicine/Medical/Clinical journals.

#### 3.4.3 Top 10 highly cited references and co-cited references

**Figure 5:**
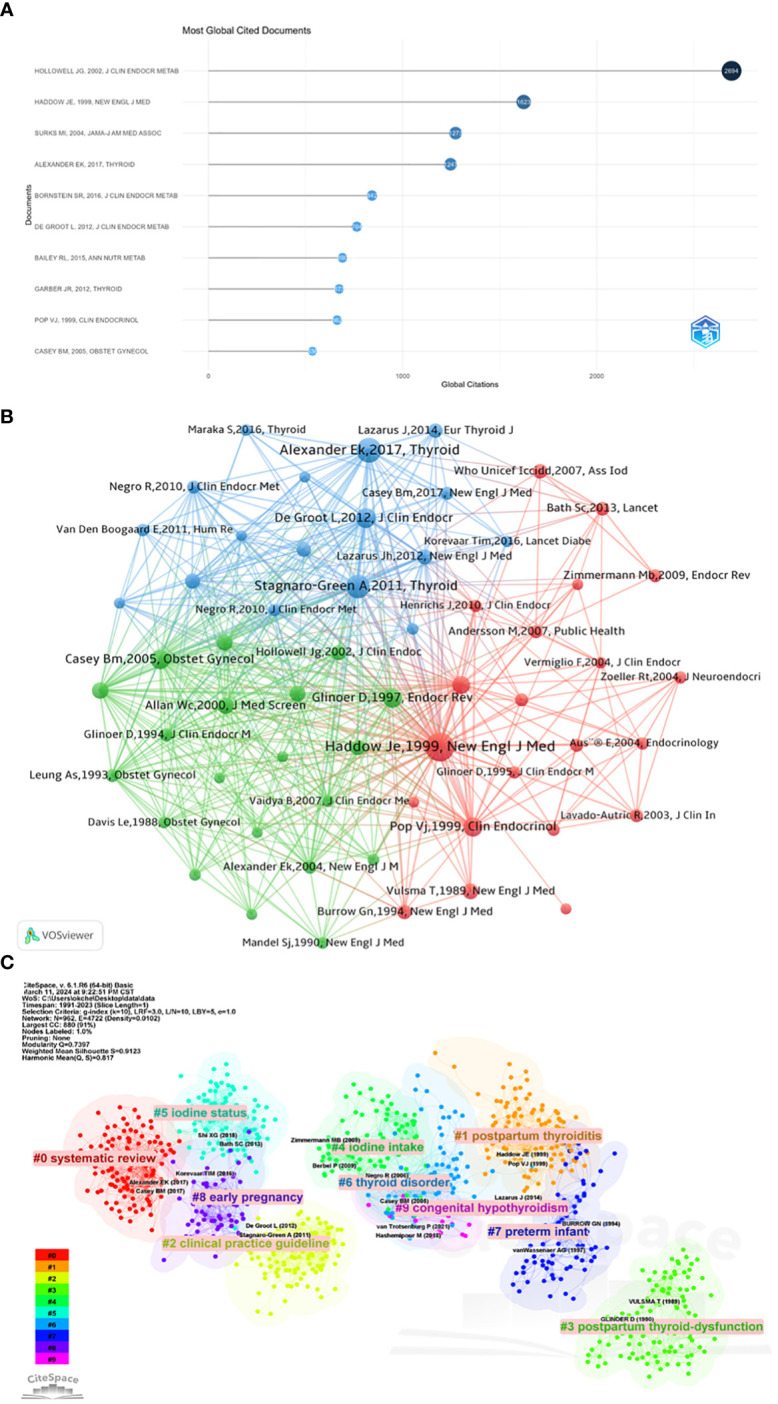
Top 10 highly cited references and co-cited references. **(A)** Top 10 most cited articles in Maternal Hypothyroidism research. **(B)** Co-citation network analysis of most cited references. **(C)** Cluster analysis of co-cited references.

Of the 4184 articles, they have been cited a total of 114378 times, with a median citation count of 10 times. **Table 6** and **Figure 5A** lists the top 10 most cited articles in the field of maternal hypothyroidism, ranging from 536 to 2694 citations. The article “Serum TSH, T (4), and thyroid antibodies in the USA population (1988 to 1994): National Health and Nutrition Examination Survey (NHANES III),” published in 2002 in the “Journal of Clinical Endocrinology & Metabolism,” stands out with the highest citation count (2694 citations). The article “2017 Guidelines of the American Thyroid Association for the Diagnosis and Management of Thyroid Disease During Pregnancy and the Postpartum,” published in 2017 in “Thyroid,” has the highest average yearly citation count (178.14).

**Table 6:** Top 10 most cited articles in Maternal Hypothyroidism research.

Title	Journal	Year of publication	Total Citations	Average per Year
Serum TSH, T(4), and thyroid antibodies in the United States population (1988 to 1994): National Health and Nutrition Examination Survey (NHANES III)	Journal Of Clinical Endocrinology & Metabolism	2002	2694	122.45
Maternal thyroid deficiency during pregnancy and subsequent neuropsychological development of the child	New England Journal Of Medicine	1999	1623	64.92
Subclinical thyroid disease - Scientific review and guidelines for diagnosis and management	Jama-Journal Of The American Medical Association	2004	1273	63.65
2017 Guidelines of the American Thyroid Association for the Diagnosis and Management of Thyroid Disease During Pregnancy and the Postpartum	Thyroid	2017	1247	178.14
Diagnosis and Treatment of Primary Adrenal Insufficiency: An Endocrine Society Clinical Practice Guideline	Journal Of Clinical Endocrinology & Metabolism	2016	842	105.25
Management of Thyroid Dysfunction during Pregnancy and Postpartum: An Endocrine Society Clinical Practice Guideline	Journal Of Clinical Endocrinology & Metabolism	2012	764	63.67
The Epidemiology of Global Micronutrient Deficiencies	Annals Of Nutrition And Metabolism	2015	690	76.67
Clinical Practice Guidelines for Hypothyroidism in Adults: Cosponsored by the American Association of Clinical Endocrinologists and the American Thyroid Association	Thyroid	2012	673	56.08
Low maternal free thyroxine concentrations during early pregnancy are associated with impaired psychomotor development in infancy	Clinical Endocrinology	1999	662	26.48
Subclinical hypothyroidism and pregnancy outcomes	Obstetrics And Gynecology	2005	536	28.21

The top 10 co-cited references in the Maternal Hypothyroidism field are listed in **Table 7**. The most co-cited article is “Maternal thyroid deficiency during pregnancy and subsequent neuropsychological development of the child” (699 citations), followed by “2017 Guidelines of the American Thyroid Association for the Diagnosis and Management of Thyroid Disease During Pregnancy and the Postpartum” (507 citations). The third most co-cited article is “Guidelines of the American Thyroid Association for the Diagnosis and Management of Thyroid Disease During Pregnancy and Postpartum” (463 citations).

**Table 7:** Top 10 co-cited references in Maternal Hypothyroidism field.

Rank	Title	Journal	Year of publication	Number of references co-cited
1	Maternal thyroid deficiency during pregnancy and subsequent neuropsychological development of the child	New England Journal of Medicine	1999	699
2	2017 Guidelines of the American Thyroid Association for the Diagnosis and Management of Thyroid Disease During Pregnancy and the Postpartum	Thyroid	2017	507
3	Guidelines of the American Thyroid Association for the Diagnosis and Management of Thyroid Disease During Pregnancy and Postpartum	Thyroid	2011	463
4	Management of Thyroid Dysfunction during Pregnancy and Postpartum: An Endocrine Society Clinical Practice Guideline	Journal of Clinical Endocrinology & Metabolism	2012	345
5	Subclinical hypothyroidism and pregnancy outcomes	Obstetrics & Gynecology	2005	311
6	The regulation of thyroid function in pregnancy: Pathways of endocrine adaptation from physiology to pathology	Endocrine Reviews	1997	310
7	Low maternal free thyroxine concentrations during early pregnancy are associated with impaired psychomotor development in infancy	Clinical Endocrinology	1999	309
8	Maternal hypothyroxinaemia during early pregnancy and subsequent child development: a 3-year follow-up study	Clinical Endocrinology	2003	262
9	Maternal thyroid deficiency and pregnancy complications: implications for population screening	Journal of Medical Screening	2000	230
10	Overt and subclinical hypothyroidism complicating pregnancy	Thyroid	2002	220

It is noteworthy that five articles among the top 10 co-cited references also appear in the top 10 highly cited articles. These articles, including “Maternal thyroid deficiency during pregnancy and subsequent neuropsychological development of the child,” “2017 Guidelines of the American Thyroid Association for the Diagnosis and Management of Thyroid Disease During Pregnancy and the Postpartum,” “Management of Thyroid Dysfunction during Pregnancy and Postpartum: An Endocrine Society Clinical Practice Guideline,” “Subclinical hypothyroidism and pregnancy outcomes,” and “Low maternal free thyroxine concentrations during early pregnancy are associated with impaired psychomotor development in infancy,” underscore their paramount importance and influence in the field. Widely acknowledged and frequently cited, these articles collectively serve as cornerstone contributions to Maternal Hypothyroidism research.

**Figure 5B** displays the co-citation network analysis of the most frequently cited references. The size of nodes represents the total number of citations of references, and the thickness of lines connecting two nodes represents the strength of co-citation relationships. The color of nodes represents the clusters to which the articles belong. Each reference in **Figure 5B** is represented by its authors, publication year, and journal. VOSviewer parameters were set as follows: Method (association strength), Minimum number of citations of a cited reference: 100. We retrieved 77162 cited references, of which 58 meet the threshold. **Figure 5B** shows three clusters: red, blue, and green. The first cluster (red) primarily focuses on the prevention and control of iodine deficiency in pregnant and lactating women, and how this deficiency affects maternal thyroid function and neurodevelopment of children. For example, the study by Haddow, et al. (20) focuses on “Maternal thyroid deficiency during pregnancy and subsequent neuropsychological development of the child.” The second cluster (blue) mainly focuses on the diagnosis, management, and impact on maternal and infant health of maternal hypothyroidism during pregnancy. Alexander, E.K., et al., participated in the 2017 American Thyroid Association guidelines for the diagnosis and treatment of thyroid diseases during pregnancy and postpartum, providing valuable references for healthcare professionals to accurately diagnose and manage thyroid diseases during pregnancy and postpartum. Cluster 3 (green) primarily focuses on the impact and management of clinical hypothyroidism and subclinical hypothyroidism during pregnancy. It covers a range of topics from the regulation of thyroid function during pregnancy to the effects of hypothyroidism on pregnant women and fetuses, to screening and management strategies for these conditions.

We further employed CiteSpace for visualizing co-cited references, as illustrated in **Figure 5C**. The time span was set from 1991 to 2023, with a time slice of 1 year, focusing on reference nodes. We applied the selection criterion (g-index k=10). The resulting network comprises 962 nodes and 4722 links. The top 10 largest clusters include: systematic review, clinical practice guideline, postpartum thyroid dysfunction, iodine intake, iodine status, thyroid disorder, preterm infant, thyroid disorder, congenital hypothyroidism.

### 3.5 Keyword analysis

**Figure 6:**
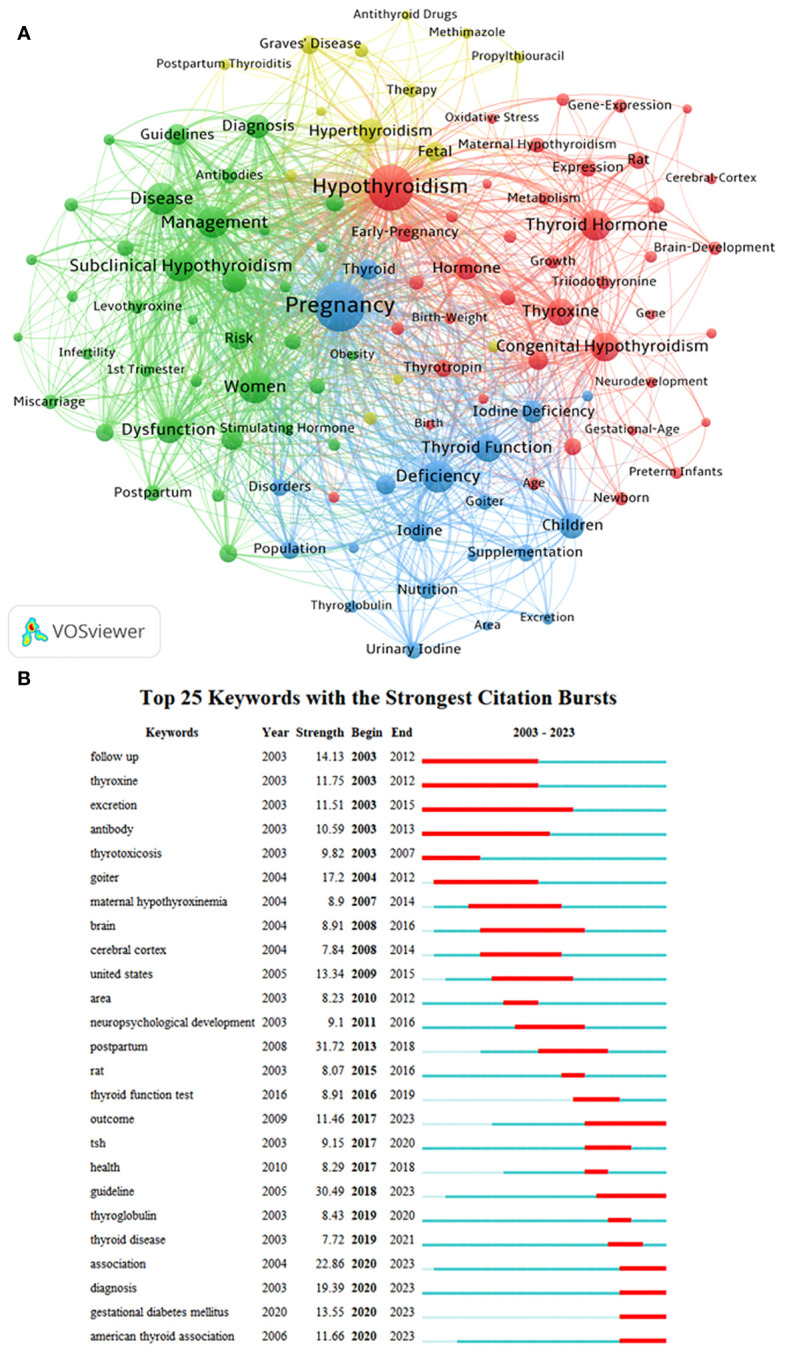
Analysis of keywords associated with Maternal Hypothyroidism. **(A)** Clustering of key terms. **(B)** Burst detection of keywords.

Utilizing VOSviewer with parameters set to Method (Association Strength) and a minimum keyword occurrence of 50, a total of 9952 keywords were identified, with 111 meeting the specified threshold. The keyword co-occurrence network map (**Figure 6A**) illustrates the varying frequencies of simultaneous keyword appearances. Among the top 50 keywords, four distinct clusters were identified based on co-occurrence rates. In **Figure 6A**, the red cluster, led by “Hypothyroidism,” primarily encompasses keywords such as “Thyroid Hormone,” “Congenital Hypothyroidism,” “Thyroxine,” “Hormone,” “Hypothyroxinemia,” “Early-Pregnancy,” “Expression,” “Thyrotropin,” and “Infant.” The green cluster predominantly includes keywords like “Women,” “Disease,” “Subclinical Hypothyroidism,” “Management,” “Dysfunction,” “Association,” “Diagnosis,” “Risk,” “Prevalence,” and “Guidelines.” The blue cluster comprises keywords such as “Pregnancy,” “Deficiency,” “Thyroid Function,” “Children,” “Iodine Deficiency,” “Iodine,” “Thyroid,” “Pregnant Women,” “Population,” and “Nutrition.” Lastly, the yellow cluster includes keywords like “Hyperthyroidism,” “Fetal,” “Graves’ Disease,” “Therapy,” “Thyrotoxicosis,” “Follow-Up,” “Propylthiouracil,” “Methimazole,” and “Thyroid Disease.”

The burst detection of keywords over the past 20 years reveals the most impactful terms in different periods (**Figure 6B**). “Postpartum” emerged as a prominent research focus from 2013 to 2018, displaying the highest burst strength (31.72). Following closely are bursts related to “Guideline” (30.49), “Association” (22.86), “Diagnosis” (19.39), “Goiter” (17.2), “Follow-Up” (14.13), “Gestational Diabetes Mellitus” (13.55), “United States” (13.34), “Thyroxine” (11.75), and “American Thyroid Association” (11.66). Examining the start times of these bursts indicates that terms like “Follow-Up,” “Thyroxine,” “Excretion,” “Antibody,” “Thyrotoxicosis,” and “Goiter” drew attention over a decade ago. On the other hand, “Outcome,” “Guideline,” “Gestational Diabetes Mellitus,” “American Thyroid Association,” “Association,” and “Diagnosis” represent recent frontiers in Maternal Hypothyroidism research.

**Figure 7:**
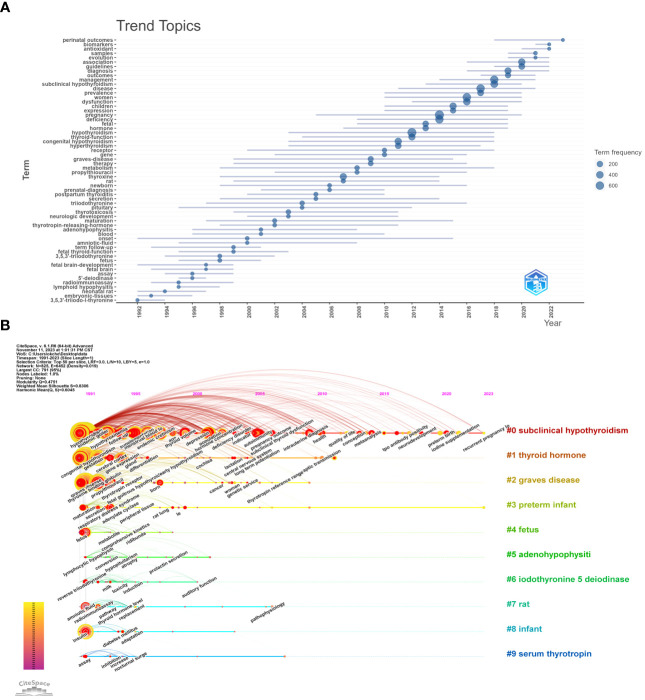
Trend analysis and Timeline viewer of Maternal Hypothyroidism topics. **(A)** Trend topics analysis. **(B)** Timeline viewer related to Maternal Hypothyroidism.

We further conducted Trend Topics analysis using the R language bibliometrix package to perform time series analysis of keywords in the Maternal Hypothyroidism literature dataset, aiming to identify evolving research trends and topics over time. In **Figure 7A**, the plotted data illustrates the evolution of various topics over time. Each line represents the lifecycle of a topic from emergence, peak, to decline. It is noteworthy that some topics have short spans (thin lines), indicating rapid fluctuations in interest, while others exhibit longer relevancy (long lines). Top Topics from 1992-2002: Onset, 3,5,3’-triiodo-L-thyronine, Neonatal rat, Fetal brain-development, Embryonic-tissues. From 2002-2012, Top Topics: Thyroxine, Graves-disease, Therapy, Thyrotoxicosis, Metabolism. From 2012-2022, Top Topics: Pregnancy, Hypothyroidism, Women, Disease, Deficiency. From 2022-2032, Top Topics: Association, Perinatal outcomes, Diagnosis, Guidelines, Evolution.

The timeline viewer in CiteSpace is also utilized to depict the evolving trends of research fields over time. Configured with parameters set to a time slice (1991–2023), one year per slice, node type (keywords), selection criteria (top N=50), and no pruning, it generated a network with 825 nodes, 6452 connections, and a density of 0.019 (**Figure 7B**). The top 10 clusters include “Subclinical Hypothyroidism,” “Thyroid Hormone,” “Graves Disease,” “Preterm Infant,” “Fetus,” “Adenohypophysiti,” “Iodothyronine 5 Deiodinase,” “Rat,” “Infant,” and “Serum Thyrotropin.” The keywords prevalent in 1991 and 2000 revolved around “Pregnancy,” “Hypothyroidism,” “Disease,” “Women,” “Deficiency,” “Subclinical Hypothyroidism,” “Management,” “Thyroid Hormone,” “Congenital Hypothyroidism,” and “Thyroid Function.” In the years 2020-2022, emerging keywords include “Gestational Diabetes Mellitus,” “Urinary Iodine Concentration,” “Preterm Birth,” “Sample,” “Iodine Supplementation,” “Obesity,” “Physiology,” “Recurrent Pregnancy Loss,” “Trimester,” “Small for Gestational Age,” and “Perinatal Outcome.”

## 4 Discussion

### 4.1 Main findings

**Figure 8:**
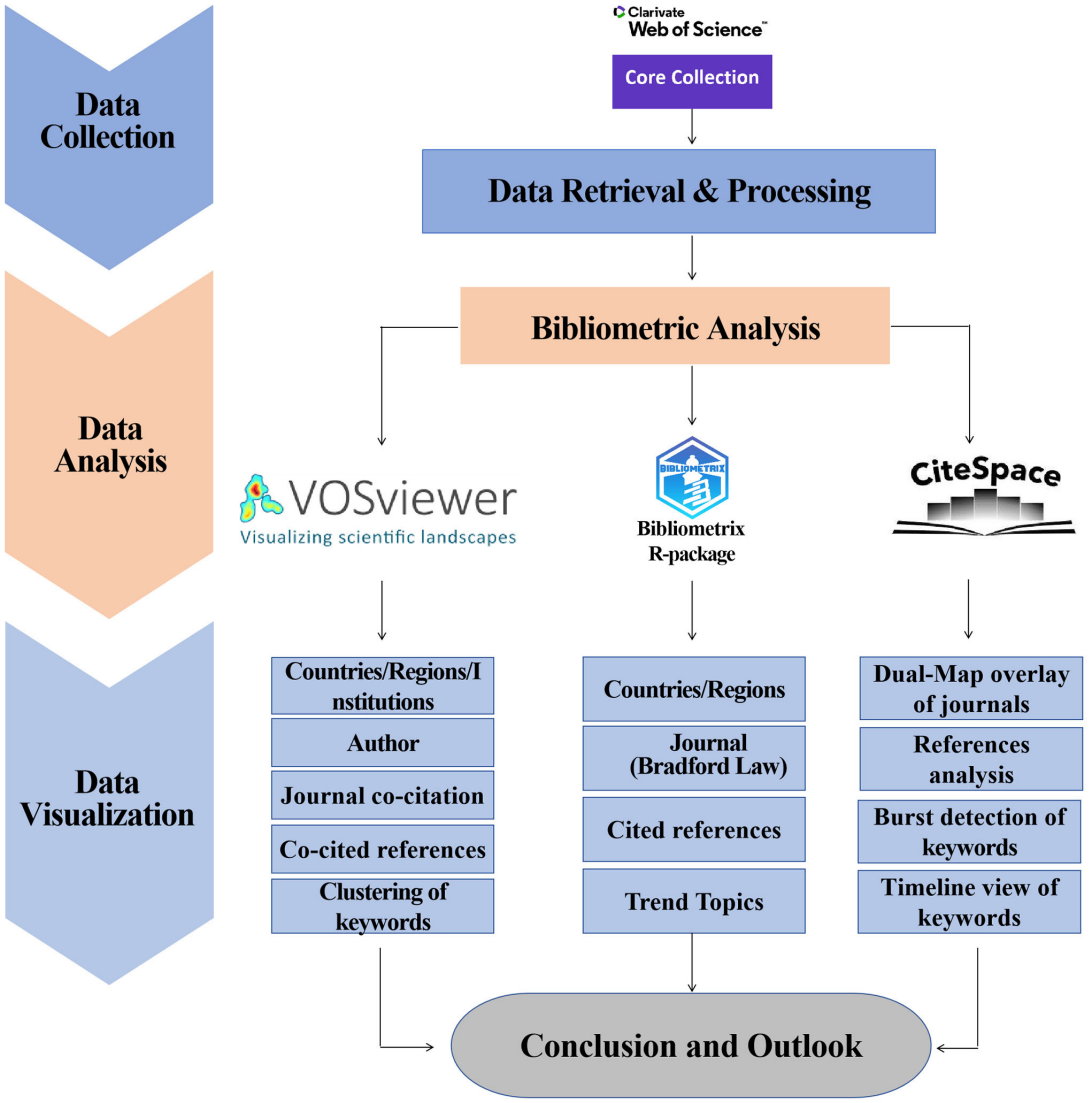
Schematic diagram of the bibliometric analyses performed.

In this study, we employed CiteSpace, VOSviewer, and bibliometrix analysis software to examine the literature related to Maternal Hypothyroidism, providing a comprehensive review of research achievements and advancements. As shown in the schematic diagram in **Figure 8**, we conducted a quantitative analysis of basic information such as annual publication count, country, institution, author, discipline, and journal. The analysis covered 4,184 articles published since 1991, accumulating a total citation count of 114,378, indicating a rising trend in the field. Researchers from 113 countries/regions across six continents participated in Maternal Hypothyroidism studies, underscoring the global significance and relevance of this topic to public health worldwide.

The leading nations in Maternal Hypothyroidism research were identified as the USA, China, England, and the Netherlands. Among the top 10 institutions, three were from the USA and two from the Netherlands, with China Medical University having the highest number of published papers and Harvard University receiving the highest citation count. Collaborations between countries and institutions were notably close, fostering a conducive environment for academic collaboration and facilitating further research in the field of Maternal Hypothyroidism.

Among the top 10 authors, Professor Teng, Weiping, with 52 published papers (1.243%), emerged as the most prolific, followed by Shan, Zhongyan (51 papers, 1.219%) and Pearce, Elizabeth N. (50 papers, 1.195%). This indicates a sustained and in-depth commitment to Maternal Hypothyroidism research by these three authors. Professors Peeters, Robin P. and Visser, Theo J. from Erasmus University Medical Center had the highest H-indices, signifying their significant contributions to the field. The collaboration between Professor Teng, Weiping, and Professor Shan, Zhongyan, focused on exploring the associations between maternal thyroid health during pregnancy in China and its correlation with offspring health. They investigated the potential impact and underlying mechanisms of maternal hypothyroidism, nutritional iodine (21), iron (22, 23), environmental factors (polybrominated diphenyl ethers) (24), and autoimmunity (23) on thyroid function during pregnancy, miscarriage (25), offspring neurodevelopment (26–28), cognition (29), and related disease mechanisms. They provided valuable insights for maternal health during pregnancy in China (30, 31).

Professor Pearce, Elizabeth N. concentrated on the diagnosis, management, and iodine nutrition of thyroid diseases during pregnancy and postpartum. She actively participated in formulating the 2017 American Thyroid Association guidelines for the diagnosis and management of thyroid diseases during pregnancy and postpartum, contributing to the standardization and regulation of this field (32). Her extensive research covered global and regional iodine nutrition status (33), including pregnant women (34) and iodine nutrition during breastfeeding (35). She investigated the impact of environmental pollutants such as perchlorate and thiocyanate on thyroid function in pregnant women (36). Furthermore, she explored the relationships between maternal thyroid function and complications during pregnancy, such as preterm birth (13), pregnancy-induced hypertension, and preeclampsia (37). Professor Pearce, Elizabeth N. proposed a series of clinical practice and research recommendations for thyroid diseases during pregnancy and lactation, providing crucial references for healthcare professionals and researchers.

Professors Visser, Theo J., and Peeters, Robin P., collaborated extensively, focusing on the association between maternal thyroid function and pregnancy complications [preeclampsia (11, 38), pregnancy-induced hypertension (10), and preterm birth (13, 39)], placenta (40), and offspring health [birth weight (41), attention deficit/hyperactivity disorder (42), cognition (43), intelligence and brain morphology (44), neurodevelopment, and behavioral issues (45, 46)]. Their research delved into various factors influencing thyroid function during pregnancy, such as thyroid autoimmunity (47), iodine levels (48), and their effects on the mother and fetus. They conducted studies on the reference range of childhood thyroid function and reviewed the determinants of thyroid function through literature and prospective cohort studies (49). Their research guided the monitoring of thyroid function and iodine intake in pregnant women, aiming to improve pregnancy outcomes and promote healthy offspring development. They made significant contributions to the diagnosis, management, prevention, and treatment of thyroid diseases during pregnancy and postpartum.

Further, we conducted co-citation analysis using the cited authors as units of analysis. The top five authors with the most citations are Glinoer, Daniel; Zimmermann, Mb; Haddow, Je; Stagnaro-Green, A; and Negro, R. Professor Glinoer’s research primarily focuses on the intersection of thyroid function, pregnancy, and reproductive health, including thyroid regulation and dysfunction during pregnancy and their management (50–52), autoimmune thyroid diseases and reproductive health (53), iodine nutrition and thyroid health (54), thyroid hormones and pregnancy outcomes, interventions, and recommendations for thyroid disease management during pregnancy (55, 56). Their work aims to improve clinical practice (57), particularly in the context of thyroid-related issues. Professor Zimmermann’s research primarily focuses on nutritional deficiencies, particularly iodine (58, 59) and iron (60), and their impact on public health (61), focusing on nutritional status assessments in pregnant women, infants, and children in different geographical settings (62, 63). His research deepens the understanding of the impact of iodine and iron deficiencies on public health, informs strategies to alleviate these deficiencies, and influences policies and guidelines to improve global nutrition status and health outcomes. Professor Haddow’s research primarily focuses on prenatal screening and maternal health, particularly addressing hypothyroidism, Down syndrome, trisomy syndromes, cystic fibrosis, and the impact of environmental factors such as tobacco smoke on child development. Works related to Maternal Hypothyroidism include the effects of maternal hypothyroidism on child neurodevelopment (20, 64, 65), reference ranges and individual variations in thyroid-stimulating hormone levels in early to mid-pregnancy (66), and the importance of adequate maternal iodine intake and its impact on fetal outcomes (67, 68). Professor Stagnaro-Green, A’s research primarily focuses on thyroid diseases during pregnancy and their impact on maternal and infant health. His contributions can be categorized into several key areas: diagnosis and management of thyroid diseases during pregnancy and postpartum (69, 70), evaluating the effectiveness and cost-effectiveness of universal screening and targeted case finding for thyroid disorders in pregnant women (71), aimed at optimizing outcomes for mothers and offspring, prevalence of thyroid diseases during pregnancy (7), and impact on pregnancy complications (72–74). Professor Negro, R’s research primarily focuses on thyroid diseases during pregnancy and their management, emphasizing understanding the impact of thyroid function and autoimmune thyroid diseases on pregnancy outcomes. He has made significant contributions to establishing and updating guidelines for the diagnosis and management of thyroid diseases during pregnancy and postpartum (69, 75, 76). This includes developing clinical practice guidelines to aid in identifying, treating, and monitoring thyroid dysfunction in pregnant and postpartum women. These authors hold significant positions in the co-citation network and collaborate closely.

In terms of disciplinary distribution, as outlined in **Table 5**, more than one-third of the articles on Maternal Hypothyroidism have been published in the field of Endocrinology Metabolism (1,531 articles, 36.592%). The journal “Thyroid” has emerged as the primary outlet for articles related to Maternal Hypothyroidism, with 233 publications (5.57%), followed by the “Journal of Clinical Endocrinology & Metabolism” (202 publications, 4.83%), and “Clinical Endocrinology” (116 publications, 2.77%). Among the top 10 journals in the field of Maternal Hypothyroidism, six are positioned within the JCR Q1 zone. Analyzing the distribution of literature sources is instrumental in identifying core journals for the publication of Maternal Hypothyroidism-related papers, aiding future scholars in selecting appropriate outlets for their research. **Figure 4C**, presenting the dual-map overlay of journals in Molecular Biology Genetics and Health Nursing Medicine research, indicates that studies in Maternal Hypothyroidism are not only frequently cited in Molecular/Biology/Immunology journals but also extensively referenced in Medicine/Medical/Clinical journals. This implies that research on maternal hypothyroidism involves in-depth exploration at the molecular and biological levels and is directly relevant to medical, healthcare, and clinical practices. This comprehensiveness likely renders these studies appealing to scholars and professionals in different fields, resulting in frequent citations across various journals. The majority of the top 10 cited literature predominantly revolves around clinical practice guidelines, primarily because these guidelines provide specific treatment protocols and decision support for healthcare practitioners. Grounded in extensive research and expert consensus, these guidelines are widely acknowledged, authoritative, and consequently, extensively cited.

The analysis of high-frequency keywords and emergent terms reflects the hotspots and frontiers of a specific research field. Through keyword co-occurrence analysis, we identified the primary directions and hotspots of Maternal Hypothyroidism, revealing the development and evolution of its thematic structure. VOSviewer’s keyword clustering analysis ultimately yielded four clusters represented by distinct colors:

The red cluster’s keywords predominantly focus on the association between maternal hypothyroidism in pregnant women and offspring birth weight, intelligence quotient, attention-deficit/hyperactivity disorder (ADHD), cognition, intelligence, brain morphology, neural development, and behavioral issues. Mechanisms, physiological processes, and environmental exposures are also implicated. Insufficient maternal thyroid hormones may lead to intrauterine growth retardation, potentially resulting in low birth weight (77). Thyroid hormones play a crucial role in fetal brain development, and maternal hypothyroidism may impact normal development, increasing the risk of intellectual disabilities in offspring (78), diminished learning abilities (79), and other neurological issues. Some studies suggest a link between maternal hypothyroidism and an increased risk of autism (80), ADHD (81), and other neurodevelopmental problems in offspring. Hypothyroidism during pregnancy may elevate the risk of metabolic disorders in children (82). These advancements underscore the potential impact of Maternal Hypothyroidism on various aspects of offspring health. Ongoing research aims to comprehensively understand this relationship, providing more accurate guidance for clinical practice and prenatal management.

The green cluster’s co-occurring keywords center around the diagnosis, management, guidelines, treatment, risk assessment, autoimmune aspects, and various types of pregnancy complications related to maternal hypothyroidism. These complications include preterm birth, gestational hypertension, preeclampsia/eclampsia, gestational diabetes, miscarriage, and placental abruption. Preterm birth is a common complication of maternal hypothyroidism (83). Studies indicate a close association between subclinical hypothyroidism, isolated hypothyroidism, positive thyroid peroxidase antibodies (TPOAb), and the risk of preterm birth. Isolated hypothyroidism and positive TPOAb are more significantly linked to severe preterm birth (13). Furthermore, hypothyroidism may lead to metabolic abnormalities and cardiovascular dysfunction, increasing the risk of preeclampsia/eclampsia. Dominant hypothyroidism is significantly correlated with severe preeclampsia risk (84). Subclinical hypothyroidism is associated with a higher risk of preeclampsia (37), whereas isolated hypothyroidism or positive TPOAb shows no clear association with gestational hypertension or preeclampsia (11). Some studies suggest that the risk of preeclampsia-eclampsia in women with isolated hypothyroidism increases with the severity of hypothyroidism (85). Hypothyroidism also increases the risk of gestational diabetes (86). Increased thyroid-stimulating hormone levels (87) and subclinical hypothyroidism are associated with an increased risk of gestational diabetes (88). The early levels of free T4 are associated with gestational diabetes, and as the levels increase, the incidence of gestational diabetes decreases (12). Miscarriage risk is also associated with thyroid dysfunction. Women with subclinical hypothyroidism and thyroid autoimmunity have an increased risk of miscarriage during weeks 4-8 of pregnancy, especially those with both conditions, experiencing higher risks and earlier gestational ages (25). Placental abruption, a severe pregnancy complication, is associated with subclinical hypothyroidism and isolated maternal hypothyroidism (89). In 2005, Casey et al. found that at 20 weeks of pregnancy, subclinical hypothyroidism patients had a threefold increase in the risk of placental abruption and nearly a twofold increase in the risk of preterm birth (90). Studies emphasize autoimmune thyroid disease (AITD), particularly Hashimoto’s disease, which is often accompanied by the production of thyroid antibodies. Pregnant women with AITD have an increased postpartum demand for levothyroxine compared to the pre-pregnancy period (91). Researchers strive to optimize treatment strategies during pregnancy in cases of AITD, addressing how to maintain optimal thyroid function and reduce adverse effects on both patients and infants.

Research on Maternal Hypothyroidism focuses on early screening and diagnosis of thyroid function in pregnant women to ensure the timely detection and management of potential thyroid dysfunction (92). Through large-scale studies, researchers have conducted a more detailed analysis of the dynamic changes in thyroid hormone levels during pregnancy to better understand the fluctuations in thyroid hormones (93). Researchers have compared and analyzed reference values for thyroid hormones during pregnancy in different regions, populations, and gestational weeks to establish more adaptive reference ranges for various populations (94–97). Attention has been given to the relationship between thyroid hormone levels during pregnancy and pregnancy outcomes (such as preterm birth and low birth weight) (98), aiming to establish more suitable reference ranges (32). This ensures a more accurate reflection of situations that pose risks to maternal and infant health. Additionally, researchers have established thyroid hormone reference ranges applicable to infants in different seasons, regions, races, and circumstances, including preterm birth, through large-scale cross-sectional and longitudinal studies (49, 99–101). These efforts aim to ensure a more accurate reflection of the normal physiological state of this age group. Studies emphasize the correlation between thyroid hormone levels in infants and aspects such as intelligence and growth (102), contributing to a deeper understanding of the impact of thyroid hormones on child development. In summary, these studies contribute to a more accurate determination of normal thyroid hormone ranges during pregnancy and infancy in different contexts. They underscore the importance of establishing accurate thyroid hormone reference values for clinical management, especially in high-risk pregnant women, to detect and address potential thyroid issues early and provide more precise assessment and management for the health of both pregnant women and fetuses.

For a long time, the safety of thyroid hormone replacement therapy has been a focal point. Pregnant women with hypothyroidism need to increase levothyroxine (T4) dosage (an average increase of 50%), with this increased dosage mainly occurring in the first half of pregnancy and stabilizing by the 16th week of pregnancy (103). However, both high and low maternal FT4 concentrations are associated with lower child IQ, suggesting that overcorrection of maternal hypothyroidism may itself have adverse effects (44). In recent years, progress has been made regarding the safety of thyroid hormone replacement therapy, including studies on different dosages (104, 105). Research also emphasizes adjusting thyroid hormone dosage and administration methods based on the specific circumstances of patients (106). Additionally, some studies are dedicated to developing new types of thyroid hormone medications to improve the drawbacks of traditional thyroid hormone replacement therapy, such as enhancing formulations (107). Several large clinical trials have confirmed the effectiveness of thyroid hormone replacement therapy in the treatment of hypothyroidism, making it the standard treatment method (108, 109). These studies demonstrate that thyroid hormone replacement therapy significantly improves patients’ quality of life and alleviates symptoms associated with hypothyroidism. Recent research has also explored the combined use of levothyroxine (T4) and triiodothyronine (T3), indicating that monotherapy with levothyroxine (T4) is the standard treatment, and there is not enough evidence to widely recommend combination therapy (110).

Research has also delved into the management and treatment strategies for hypothyroidism during pregnancy to ensure the maintenance of appropriate thyroid hormone levels and reduce the risk of adverse effects on both mother and infant (111). Updates and revisions to clinical practice guidelines provide more accurate and up-to-date diagnostic and treatment recommendations, enabling healthcare professionals to better manage hypothyroidism during pregnancy (32).

The co-occurrence clustering in the blue region highlights various themes in the research on Maternal Hypothyroidism, including thyroid function during pregnancy, iodine deficiency, potential impacts on offspring health, and regional disparities. Iodine is a crucial element for thyroid hormone synthesis (112), and some studies emphasize the importance for pregnant women to maintain sufficient iodine intake to prevent thyroid issues caused by iodine deficiency (32). Severe iodine deficiency in pregnant women is associated with adverse pregnancy outcomes (113, 114). Additionally, iodine plays a critical role in the development of the fetal brain and nervous system (21). Iodine deficiency in pregnant women and fetuses can have detrimental effects on cognitive function in the offspring (115, 116). Both insufficient (112) and excessive (117) iodine intake may lead to maternal hypothyroidism. Researchers have explored the relationship between iodine intake and maternal thyroid hormone levels, with adequate iodine intake considered helpful in maintaining normal thyroid function (118, 119) Multiple studies focus on monitoring postpartum mothers’ iodine nutrition status (120–122), aiding in the formulation of iodine supplementation strategies and providing guidance on iodine intake recommendations. Overall, researchers have conducted in-depth investigations into the relationship between iodine and Maternal Hypothyroidism to promote effective management of the health of pregnant and postpartum women. These studies contribute to guiding public health policies and providing more comprehensive advice for antenatal care.

The co-occurrence clustering in the yellow region points to various themes in the research on maternal thyroid dysfunction, including hyperthyroidism-related disorders. These themes encompass “treatment methods, fetal effects”, “epidemiological data”, and “therapeutic drugs”, focusing on understanding the impact of maternal thyroid dysfunction on maternal and infant health and providing relevant guidance and recommendations for clinical practice. Keywords such as “Hyperthyroidism”, “Thyrotoxicosis”, “Graves’ Disease”, “Postpartum Thyroiditis”, “Antithyroid Drugs” involve maternal thyroid dysfunction and corresponding treatment methods like antithyroid drugs (such as methimazole, propylthiouracil). “Fetal”, “Mothers”, “Human Chorionic-Gonadotropin” indicate the potential effects of maternal thyroid dysfunction on the fetus and the related physiological mechanisms. “Epidemiology”, “Follow-Up” suggest research on the epidemiology of maternal thyroid dysfunction and patient follow-up.

Subsequently, we identified the research focal points and frontiers of Maternal Hypothyroidism through an analysis of the Timeline viewer related to Maternal Hypothyroidism and the top 25 keywords with the most substantial citation bursts. The primary research focal points are as follows (1):Pregnancy and postpartum outcomes: Research places emphasis on postpartum and pregnancy outcomes, considering the impact of thyroid disorders on maternal and infant health (2).Diagnosis and guidelines: The roles of diagnosis and guidelines are pivotal in managing thyroid disorders during pregnancy (3).Thyroid hormones and function: The influence of thyroxine concentration and thyroid dysfunction (thyrotoxicosis, hypothyroidism) on maternal health (4).United States research and collaborations: United States research and international cooperation (meta-analysis, American Thyroid Association) hold significant positions in the studies (5).Rodent models and physiology: Keywords such as adult rat and physiology are commonly observed, underscoring the importance of animal models in research (6).Thyroid disorders and autoimmune diseases: Crucial aspects of the research encompass thyroid disorders, including autoimmune thyroiditis and Graves’ disease. The forefront trends include (1):Neurological system and cognitive development: Research delves deeper into the relationship between pregnancy, thyroid dysfunction, and the brain, as well as neuropsychological development (2).Biomarkers and health outcomes: Biomarkers such as antibodies and thyroglobulin play a significant role in disease prognosis and health outcomes (3).Models and brain regions: Key areas such as the cerebral cortex, dentate gyrus, and adult rodent models hold substantial importance in research (4).Gestational diabetes and health: There is a close association between gestational diabetes mellitus and health in Maternal Hypothyroidism research.

Further Trend Topics analysis revealed significant evolution in the hot topics within the field of Maternal hypothyroidism from 1992 to 2032. Early years (1992–2002) primarily focused on basic biology such as 3,5,3’-Triiodo-L-thyronine, neonatal rats, and fetal brain development. Subsequently (2002–2012), research shifted towards specific health issues like thyroid hormones, Graves’ disease, and their treatments. In recent years (2012–2022), women’s health issues such as pregnancy and hypothyroidism have become research hotspots. Looking ahead, there is expected emphasis on diagnostics, guideline development, and the association between diseases and antioxidants. This evolution highlights the dynamism of scientific research and its response to new technologies and societal needs. As scientific exploration deepens, understanding of health and diseases is progressing towards a more comprehensive and preventative direction.

### 4.2 Strengths and limitations

This study marks the pioneering application of bibliometric analysis to the Maternal Hypothyroidism field, providing a fresh perspective and aiding a profound understanding of its developmental trends. In contrast to traditional literature reviews, our study employed various bibliometric analysis tools, including CiteSpace, VOSviewer, and the R software package bibliometrix. The integrated use of these tools enables a comprehensive and systematic extraction and analysis of data, thereby revealing the dynamic research landscape of the Maternal Hypothyroidism field. However, limitations exist, primarily reflected in the data source being restricted to the WoSCC database, potentially leading to the oversight of relevant information in other databases. Furthermore, while bibliometric analysis can unveil the impact of literature, they often cannot independently assess the quality of each study due to factors like citation counts being influenced by time. Despite these limitations, the bibliometric analysis employed in this study still offer valuable insights into the research trends of the Maternal Hypothyroidism field and provide new avenues for future research directions. To further enhance the analysis of this research field, future studies may consider integrating multiple databases and analysis methods to gain more comprehensive and in-depth insights.

## 5 Conclusion

This study conducted an in-depth bibliometric analysis of Maternal Hypothyroidism to explore thematic development and future research focal points. We provided foundational information for researchers interested in the field and identified potential collaborators. Overall, Maternal Hypothyroidism research has historically focused on thyroid function diagnosis during pregnancy, treatment guidelines, iodine nutrition, the correlation between maternal and offspring health, and postpartum aspects. Recently, the research has shifted towards gestational diabetes, iodine intake, preterm birth, obesity, and the impact of iodine supplementation on postpartum outcomes, indicating these areas as crucial directions for future research. Simultaneously, sustained attention to subclinical hypothyroidism, thyroid hormones, Graves’ disease, and related disorders will continue to be research focal points. In summary, the bibliometric analysis of Maternal Hypothyroidism underscores the sustained importance of maternal thyroid health during pregnancy and postpartum. The shift towards emerging topics like gestational diabetes, iodine intake, and preterm birth opens new directions for future research, emphasizing the multifaceted impact of Maternal Hypothyroidism on maternal and infant health.

## Author contributions

AC: Investigation, Data curation, Writing – original draft. ZL: Writing – original draft, Data curation. JZ: Writing – review & editing. XC: Writing – review & editing.
